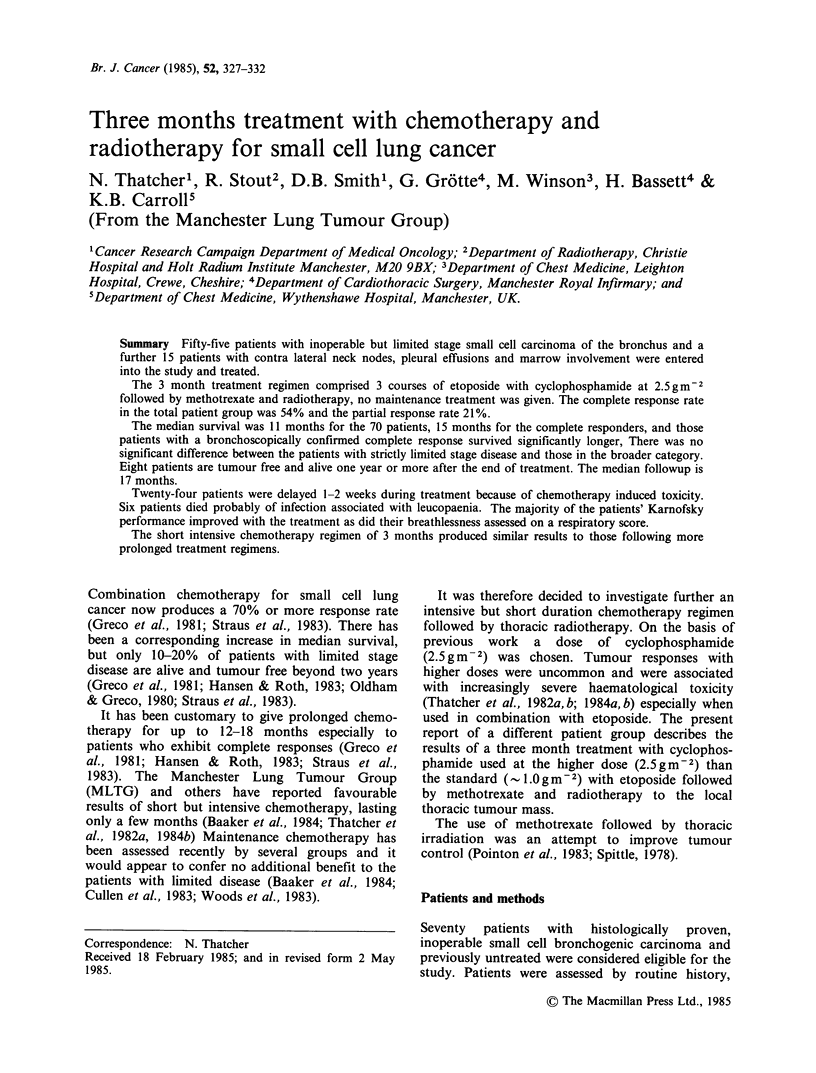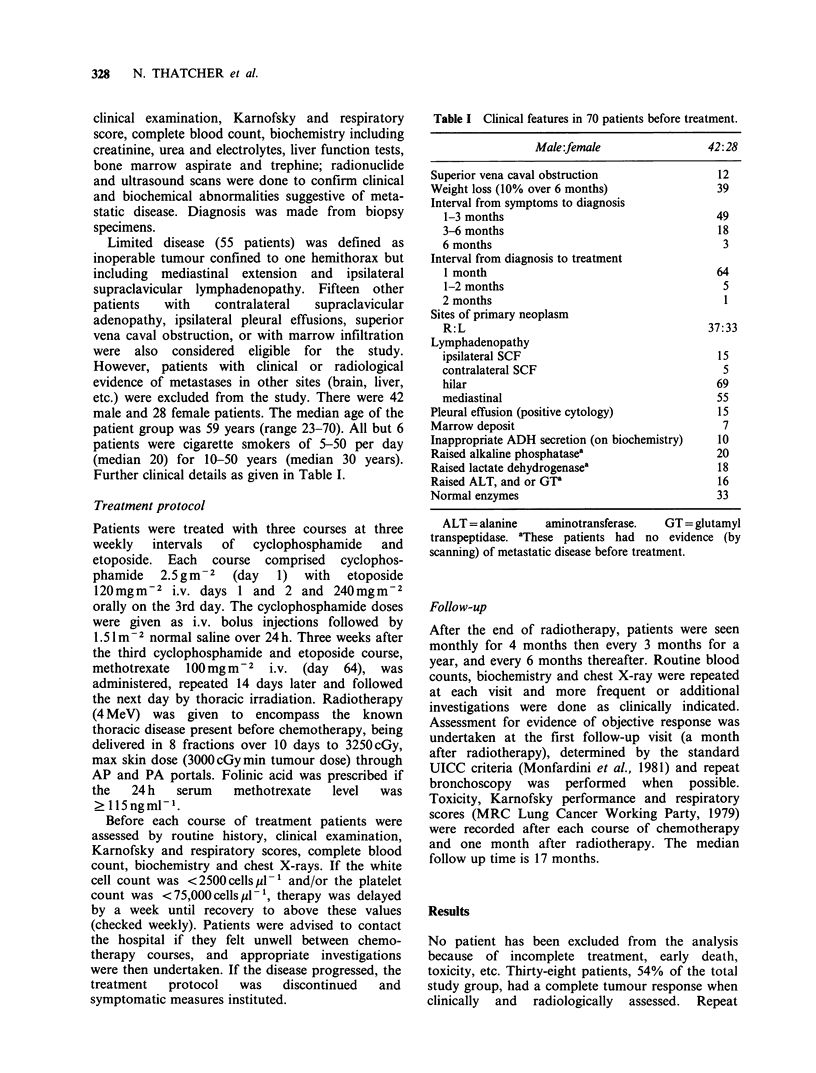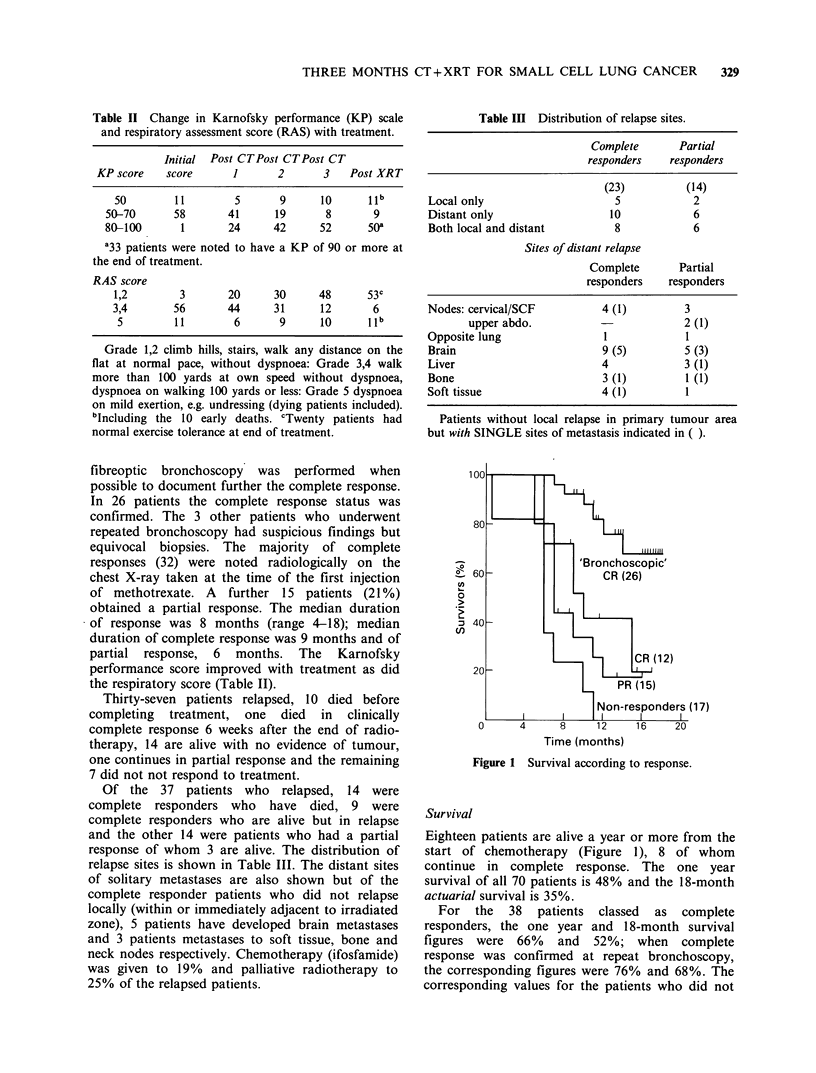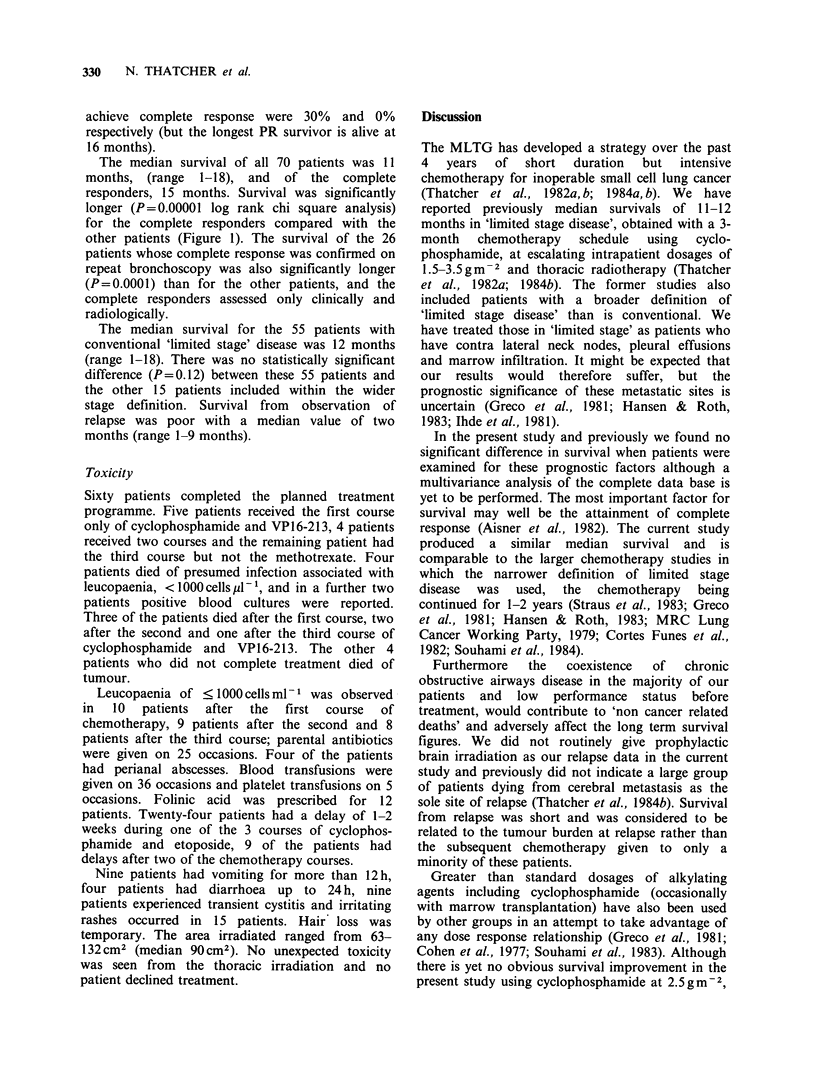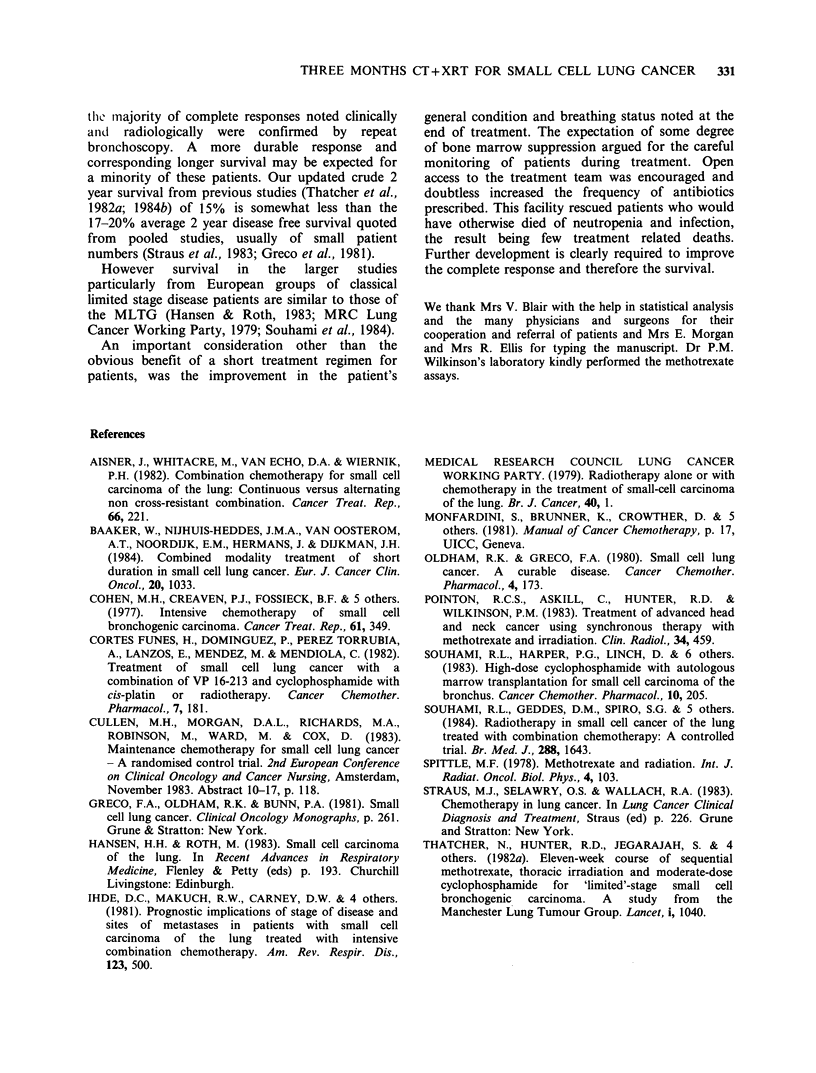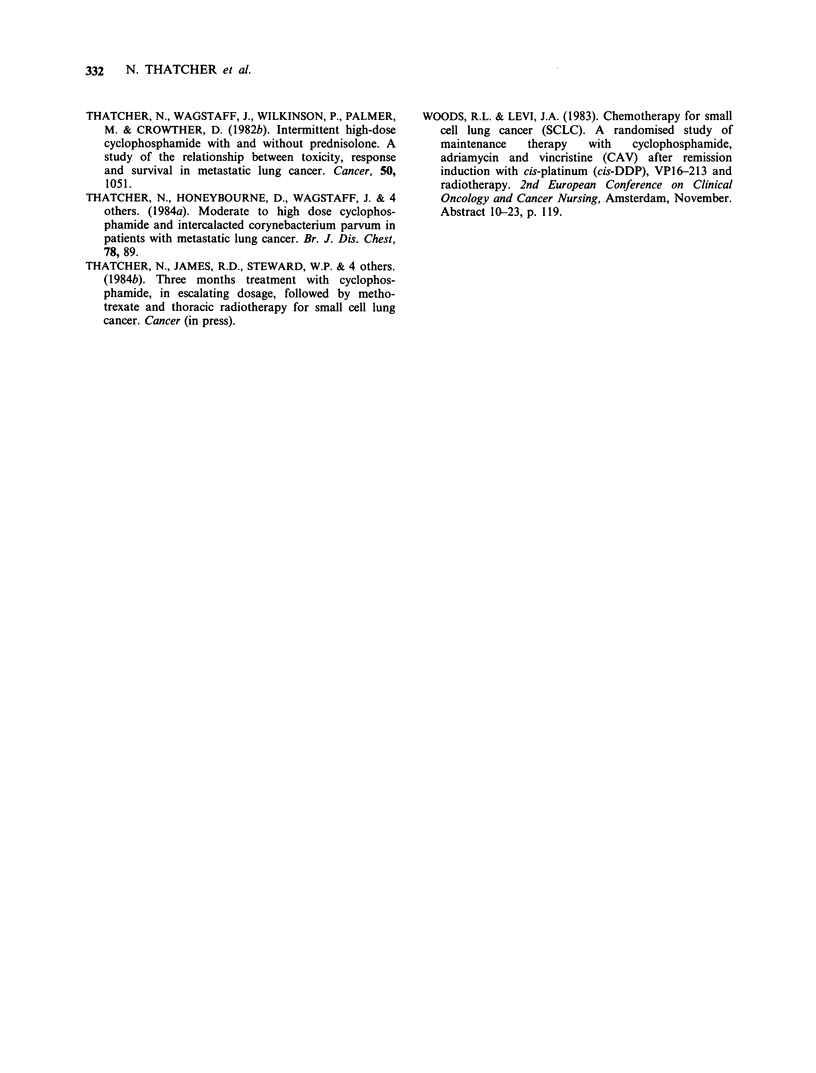# Three months treatment with chemotherapy and radiotherapy for small cell lung cancer.

**DOI:** 10.1038/bjc.1985.197

**Published:** 1985-09

**Authors:** N. Thatcher, R. Stout, D. B. Smith, G. Grötte, M. Winson, H. Bassett, K. B. Carroll

## Abstract

Fifty-five patients with inoperable but limited stage small cell carcinoma of the bronchus and a further 15 patients with contra lateral neck nodes, pleural effusions and marrow involvement were entered into the study and treated. The 3 month treatment regimen comprised 3 courses of etoposide with cyclophosphamide at 2.5 gm-2 followed by methotrexate and radiotherapy, no maintenance treatment was given. The complete response rate in the total patient group was 54% and the partial response rate 21%. The median survival was 11 months for the 70 patients, 15 months for the complete responders, and those patients with a bronchoscopically confirmed complete response survived significantly longer. There was no significant difference between the patients with strictly limited stage disease and those in the broader category. Eight patients are tumour free and alive one year or more after the end of treatment. The median followup is 17 months. Twenty-four patients were delayed 1-2 weeks during treatment because of chemotherapy induced toxicity. Six patients died probably of infection associated with leucopaenia. The majority of the patients' Karnofsky performance improved with the treatment as did their breathlessness assessed on a respiratory score. The short intensive chemotherapy regimen of 3 months produced similar results to those following more prolonged treatment regimens.


*Br. J. Cancer* (1985), **52**, 327–332

### Three months treatment with chemotherapy and radiotherapy for small cell lung cancer

N. Thatcher[1], R. Stout[2], D.B. Smith[1], G. Grötte[4], M. Winson[3], H. Bassett[4] & K.B. Carroll[5]

(From the Manchester Lung Tumour Group)

[1]*Cancer Research Campaign Department of Medical Oncology;* [2]*Department of Radiotherapy, Christie Hospital and Holt Radium Institute Manchester, M20 9BX;* [3]*Department of Chest Medicine, Leighton Hospital, Crewe, Cheshire;* [4]*Department of Cardiothoracic Surgery, Manchester Royal Infirmary; and* [5]*Department of Chest Medicine, Wythenshawe Hospital, Manchester, UK.*

**Summary** Fifty-five patients with inoperable but limited stage small cell carcinoma of the bronchus and a further 15 patients with contra lateral neck nodes, pleural effusions and marrow involvement were entered into the study and treated.

The 3 month treatment regimen comprised 3 courses of etoposide with cyclophosphamide at $2.5\,gm^{-2}$ followed by methotrexate and radiotherapy, no maintenance treatment was given. The complete response rate in the total patient group was 54% and the partial response rate 21%.

The median survival was 11 months for the 70 patients, 15 months for the complete responders, and those patients with a bronchoscopically confirmed complete response survived significantly longer, There was no significant difference between the patients with strictly limited stage disease and those in the broader category. Eight patients are tumour free and alive one year or more after the end of treatment. The median followup is 17 months.

Twenty-four patients were delayed 1–2 weeks during treatment because of chemotherapy induced toxicity. Six patients died probably of infection associated with leucopaenia. The majority of the patients' Karnofsky performance improved with the treatment as did their breathlessness assessed on a respiratory score.

The short intensive chemotherapy regimen of 3 months produced similar results to those following more prolonged treatment regimens.

Combination chemotherapy for small cell lung cancer now produces a 70% or more response rate (Greco *et al.*, 1981; Straus *et al.*, 1983). There has been a corresponding increase in median survival, but only 10–20% of patients with limited stage disease are alive and tumour free beyond two years (Greco *et al.*, 1981; Hansen & Roth, 1983; Oldham & Greco, 1980; Straus *et al.*, 1983).

It has been customary to give prolonged chemotherapy for up to 12–18 months especially to patients who exhibit complete responses (Greco *et al.*, 1981; Hansen & Roth, 1983; Straus *et al.*, 1983). The Manchester Lung Tumour Group (MLTG) and others have reported favourable results of short but intensive chemotherapy, lasting only a few months (Baaker *et al.*, 1984; Thatcher *et al.*, 1982*a*, 1984*b*) Maintenance chemotherapy has been assessed recently by several groups and it would appear to confer no additional benefit to the patients with limited disease (Baaker *et al.*, 1984; Cullen *et al.*, 1983; Woods *et al.*, 1983).

It was therefore decided to investigate further an intensive but short duration chemotherapy regimen followed by thoracic radiotherapy. On the basis of previous work a dose of cyclophosphamide ($2.5\,gm^{-2}$) was chosen. Tumour responses with higher doses were uncommon and were associated with increasingly severe haematological toxicity (Thatcher *et al.*, 1982*a,b*; 1984*a,b*) especially when used in combination with etoposide. The present report of a different patient group describes the results of a three month treatment with cyclophosphamide used at the higher dose ($2.5\,gm^{-2}$) than the standard ($\sim 1.0\,gm^{-2}$) with etoposide followed by methotrexate and radiotherapy to the local thoracic tumour mass.

The use of methotrexate followed by thoracic irradiation was an attempt to improve tumour control (Pointon *et al.*, 1983; Spittle, 1978).

### Patients and methods

Seventy patients with histologically proven, inoperable small cell bronchogenic carcinoma and previously untreated were considered eligible for the study. Patients were assessed by routine history,

Correspondence: N. Thatcher
Received 18 February 1985; and in revised form 2 May 1985.

© The Macmillan Press Ltd., 1985

clinical examination, Karnofsky and respiratory score, complete blood count, biochemistry including creatinine, urea and electrolytes, liver function tests, bone marrow aspirate and trephine; radionuclide and ultrasound scans were done to confirm clinical and biochemical abnormalities suggestive of metastatic disease. Diagnosis was made from biopsy specimens.

Limited disease (55 patients) was defined as inoperable tumour confined to one hemithorax but including mediastinal extension and ipsilateral supraclavicular lymphadenopathy. Fifteen other patients with contralateral supraclavicular adenopathy, ipsilateral pleural effusions, superior vena caval obstruction, or with marrow infiltration were also considered eligible for the study. However, patients with clinical or radiological evidence of metastases in other sites (brain, liver, etc.) were excluded from the study. There were 42 male and 28 female patients. The median age of the patient group was 59 years (range 23–70). All but 6 patients were cigarette smokers of 5–50 per day (median 20) for 10–50 years (median 30 years). Further clinical details as given in Table I.

*Treatment protocol*

Patients were treated with three courses at three weekly intervals of cyclophosphamide and etoposide. Each course comprised cyclophosphamide $2.5\,g\,m^{-2}$ (day 1) with etoposide $120\,mg\,m^{-2}$ i.v. days 1 and 2 and $240\,mg\,m^{-2}$ orally on the 3rd day. The cyclophosphamide doses were given as i.v. bolus injections followed by $1.5\,l\,m^{-2}$ normal saline over 24 h. Three weeks after the third cyclophosphamide and etoposide course, methotrexate $100\,mg\,m^{-2}$ i.v. (day 64), was administered, repeated 14 days later and followed the next day by thoracic irradiation. Radiotherapy (4 MeV) was given to encompass the known thoracic disease present before chemotherapy, being delivered in 8 fractions over 10 days to 3250 cGy, max skin dose (3000 cGy min tumour dose) through AP and PA portals. Folinic acid was prescribed if the 24 h serum methotrexate level was $\geq 115\,ng\,ml^{-1}$.

Before each course of treatment patients were assessed by routine history, clinical examination, Karnofsky and respiratory scores, complete blood count, biochemistry and chest X-rays. If the white cell count was $<2500$ cells $\mu l^{-1}$ and/or the platelet count was $<75{,}000$ cells $\mu l^{-1}$, therapy was delayed by a week until recovery to above these values (checked weekly). Patients were advised to contact the hospital if they felt unwell between chemotherapy courses, and appropriate investigations were then undertaken. If the disease progressed, the treatment protocol was discontinued and symptomatic measures instituted.

**Table I** Clinical features in 70 patients before treatment.

| *Male:female* | *42:28* |
|---|---|
| Superior vena caval obstruction | 12 |
| Weight loss (10% over 6 months) | 39 |
| Interval from symptoms to diagnosis | |
| 1–3 months | 49 |
| 3–6 months | 18 |
| 6 months | 3 |
| Interval from diagnosis to treatment | |
| 1 month | 64 |
| 1–2 months | 5 |
| 2 months | 1 |
| Sites of primary neoplasm | |
| R:L | 37:33 |
| Lymphadenopathy | |
| ipsilateral SCF | 15 |
| contralateral SCF | 5 |
| hilar | 69 |
| mediastinal | 55 |
| Pleural effusion (positive cytology) | 15 |
| Marrow deposit | 7 |
| Inappropriate ADH secretion (on biochemistry) | 10 |
| Raised alkaline phosphatase[a] | 20 |
| Raised lactate dehydrogenase[a] | 18 |
| Raised ALT, and or GT[a] | 16 |
| Normal enzymes | 33 |

ALT = alanine aminotransferase. GT = glutamyl transpeptidase. [a]These patients had no evidence (by scanning) of metastatic disease before treatment.

*Follow-up*

After the end of radiotherapy, patients were seen monthly for 4 months then every 3 months for a year, and every 6 months thereafter. Routine blood counts, biochemistry and chest X-ray were repeated at each visit and more frequent or additional investigations were done as clinically indicated. Assessment for evidence of objective response was undertaken at the first follow-up visit (a month after radiotherapy), determined by the standard UICC criteria (Monfardini *et al.*, 1981) and repeat bronchoscopy was performed when possible. Toxicity, Karnofsky performance and respiratory scores (MRC Lung Cancer Working Party, 1979) were recorded after each course of chemotherapy and one month after radiotherapy. The median follow up time is 17 months.

### Results

No patient has been excluded from the analysis because of incomplete treatment, early death, toxicity, etc. Thirty-eight patients, 54% of the total study group, had a complete tumour response when clinically and radiologically assessed. Repeat

**Table II** Change in Karnofsky performance (KP) scale and respiratory assessment score (RAS) with treatment.

| *KP score* | *Initial score* | *Post CT 1* | *Post CT 2* | *Post CT 3* | *Post XRT* |
|---|---|---|---|---|---|
| 50 | 11 | 5 | 9 | 10 | 11[b] |
| 50–70 | 58 | 41 | 19 | 8 | 9 |
| 80–100 | 1 | 24 | 42 | 52 | 50[a] |
| [a]33 patients were noted to have a KP of 90 or more at the end of treatment. | | | | | |
| *RAS score* | | | | | |
| 1,2 | 3 | 20 | 30 | 48 | 53[c] |
| 3,4 | 56 | 44 | 31 | 12 | 6 |
| 5 | 11 | 6 | 9 | 10 | 11[b] |

Grade 1,2 climb hills, stairs, walk any distance on the flat at normal pace, without dyspnoea: Grade 3,4 walk more than 100 yards at own speed without dyspnoea, dyspnoea on walking 100 yards or less: Grade 5 dyspnoea on mild exertion, e.g. undressing (dying patients included). [b]Including the 10 early deaths. [c]Twenty patients had normal exercise tolerance at end of treatment.

**Table III** Distribution of relapse sites.

| | *Complete responders* | *Partial responders* |
|---|---|---|
| | (23) | (14) |
| Local only | 5 | 2 |
| Distant only | 10 | 6 |
| Both local and distant | 8 | 6 |
| *Sites of distant relapse* | | |
| | Complete responders | Partial responders |
| Nodes: cervical/SCF | 4 (1) | 3 |
| upper abdo. | — | 2 (1) |
| Opposite lung | 1 | 1 |
| Brain | 9 (5) | 5 (3) |
| Liver | 4 | 3 (1) |
| Bone | 3 (1) | 1 (1) |
| Soft tissue | 4 (1) | 1 |

Patients without local relapse in primary tumour area but *with* SINGLE sites of metastasis indicated in ( ).

fibreoptic bronchoscopy was performed when possible to document further the complete response. In 26 patients the complete response status was confirmed. The 3 other patients who underwent repeated bronchoscopy had suspicious findings but equivocal biopsies. The majority of complete responses (32) were noted radiologically on the chest X-ray taken at the time of the first injection of methotrexate. A further 15 patients (21%) obtained a partial response. The median duration of response was 8 months (range 4–18); median duration of complete response was 9 months and of partial response, 6 months. The Karnofsky performance score improved with treatment as did the respiratory score (Table II).

Thirty-seven patients relapsed, 10 died before completing treatment, one died in clinically complete response 6 weeks after the end of radiotherapy, 14 are alive with no evidence of tumour, one continues in partial response and the remaining 7 did not not respond to treatment.

Of the 37 patients who relapsed, 14 were complete responders who have died, 9 were complete responders who are alive but in relapse and the other 14 were patients who had a partial response of whom 3 are alive. The distribution of relapse sites is shown in Table III. The distant sites of solitary metastases are also shown but of the complete responder patients who did not relapse locally (within or immediately adjacent to irradiated zone), 5 patients have developed brain metastases and 3 patients metastases to soft tissue, bone and neck nodes respectively. Chemotherapy (ifosfamide) was given to 19% and palliative radiotherapy to 25% of the relapsed patients.

**Figure 1** Survival according to response.

*Survival*

Eighteen patients are alive a year or more from the start of chemotherapy (Figure 1), 8 of whom continue in complete response. The one year survival of all 70 patients is 48% and the 18-month *actuarial* survival is 35%.

For the 38 patients classed as complete responders, the one year and 18-month survival figures were 66% and 52%; when complete response was confirmed at repeat bronchoscopy, the corresponding figures were 76% and 68%. The corresponding values for the patients who did not achieve complete response were 30% and 0% respectively (but the longest PR survivor is alive at 16 months).

The median survival of all 70 patients was 11 months, (range 1–18), and of the complete responders, 15 months. Survival was significantly longer ($P=0.00001$ log rank chi square analysis) for the complete responders compared with the other patients (Figure 1). The survival of the 26 patients whose complete response was confirmed on repeat bronchoscopy was also significantly longer ($P=0.0001$) than for the other patients, and the complete responders assessed only clinically and radiologically.

The median survival for the 55 patients with conventional 'limited stage' disease was 12 months (range 1–18). There was no statistically significant difference ($P=0.12$) between these 55 patients and the other 15 patients included within the wider stage definition. Survival from observation of relapse was poor with a median value of two months (range 1–9 months).

### *Toxicity*

Sixty patients completed the planned treatment programme. Five patients received the first course only of cyclophosphamide and VP16-213, 4 patients received two courses and the remaining patient had the third course but not the methotrexate. Four patients died of presumed infection associated with leucopaenia, $<1000$ cells $\mu l^{-1}$, and in a further two patients positive blood cultures were reported. Three of the patients died after the first course, two after the second and one after the third course of cyclophosphamide and VP16-213. The other 4 patients who did not complete treatment died of tumour.

Leucopaenia of $\leq 1000$ cells $ml^{-1}$ was observed in 10 patients after the first course of chemotherapy, 9 patients after the second and 8 patients after the third course; parental antibiotics were given on 25 occasions. Four of the patients had perianal abscesses. Blood transfusions were given on 36 occasions and platelet transfusions on 5 occasions. Folinic acid was prescribed for 12 patients. Twenty-four patients had a delay of 1–2 weeks during one of the 3 courses of cyclophosphamide and etoposide, 9 of the patients had delays after two of the chemotherapy courses.

Nine patients had vomiting for more than 12 h, four patients had diarrhoea up to 24 h, nine patients experienced transient cystitis and irritating rashes occurred in 15 patients. Hair loss was temporary. The area irradiated ranged from 63–132 $cm^2$ (median 90 $cm^2$). No unexpected toxicity was seen from the thoracic irradiation and no patient declined treatment.

### Discussion

The MLTG has developed a strategy over the past 4 years of short duration but intensive chemotherapy for inoperable small cell lung cancer (Thatcher *et al.*, 1982*a*,*b*; 1984*a*,*b*). We have reported previously median survivals of 11–12 months in 'limited stage disease', obtained with a 3-month chemotherapy schedule using cyclophosphamide, at escalating intrapatient dosages of 1.5–3.5 $g\,m^{-2}$ and thoracic radiotherapy (Thatcher *et al.*, 1982*a*; 1984*b*). The former studies also included patients with a broader definition of 'limited stage disease' than is conventional. We have treated those in 'limited stage' as patients who have contra lateral neck nodes, pleural effusions and marrow infiltration. It might be expected that our results would therefore suffer, but the prognostic significance of these metastatic sites is uncertain (Greco *et al.*, 1981; Hansen & Roth, 1983; Ihde *et al.*, 1981).

In the present study and previously we found no significant difference in survival when patients were examined for these prognostic factors although a multivariance analysis of the complete data base is yet to be performed. The most important factor for survival may well be the attainment of complete response (Aisner *et al.*, 1982). The current study produced a similar median survival and is comparable to the larger chemotherapy studies in which the narrower definition of limited stage disease was used, the chemotherapy being continued for 1–2 years (Straus *et al.*, 1983; Greco *et al.*, 1981; Hansen & Roth, 1983; MRC Lung Cancer Working Party, 1979; Cortes Funes *et al.*, 1982; Souhami *et al.*, 1984).

Furthermore the coexistence of chronic obstructive airways disease in the majority of our patients and low performance status before treatment, would contribute to 'non cancer related deaths' and adversely affect the long term survival figures. We did not routinely give prophylactic brain irradiation as our relapse data in the current study and previously did not indicate a large group of patients dying from cerebral metastasis as the sole site of relapse (Thatcher *et al.*, 1984*b*). Survival from relapse was short and was considered to be related to the tumour burden at relapse rather than the subsequent chemotherapy given to only a minority of these patients.

Greater than standard dosages of alkylating agents including cyclophosphamide (occasionally with marrow transplantation) have also been used by other groups in an attempt to take advantage of any dose response relationship (Greco *et al.*, 1981; Cohen *et al.*, 1977; Souhami *et al.*, 1983). Although there is yet no obvious survival improvement in the present study using cyclophosphamide at 2.5 $g\,m^{-2}$, the majority of complete responses noted clinically and radiologically were confirmed by repeat bronchoscopy. A more durable response and corresponding longer survival may be expected for a minority of these patients. Our updated crude 2 year survival from previous studies (Thatcher *et al.*, 1982*a*; 1984*b*) of 15% is somewhat less than the 17–20% average 2 year disease free survival quoted from pooled studies, usually of small patient numbers (Straus *et al.*, 1983; Greco *et al.*, 1981).

However survival in the larger studies particularly from European groups of classical limited stage disease patients are similar to those of the MLTG (Hansen & Roth, 1983; MRC Lung Cancer Working Party, 1979; Souhami *et al.*, 1984).

An important consideration other than the obvious benefit of a short treatment regimen for patients, was the improvement in the patient's general condition and breathing status noted at the end of treatment. The expectation of some degree of bone marrow suppression argued for the careful monitoring of patients during treatment. Open access to the treatment team was encouraged and doubtless increased the frequency of antibiotics prescribed. This facility rescued patients who would have otherwise died of neutropenia and infection, the result being few treatment related deaths. Further development is clearly required to improve the complete response and therefore the survival.

We thank Mrs V. Blair with the help in statistical analysis and the many physicians and surgeons for their cooperation and referral of patients and Mrs E. Morgan and Mrs R. Ellis for typing the manuscript. Dr P.M. Wilkinson's laboratory kindly performed the methotrexate assays.

### References

AISNER, J., WHITACRE, M., VAN ECHO, D.A. & WIERNIK, P.H. (1982). Combination chemotherapy for small cell carcinoma of the lung: Continuous versus alternating non cross-resistant combination. *Cancer Treat. Rep.*, **66**, 221.

BAAKER, W., NIJHUIS-HEDDES, J.M.A., VAN OOSTEROM, A.T., NOORDIJK, E.M., HERMANS, J. & DIJKMAN, J.H. (1984). Combined modality treatment of short duration in small cell lung cancer. *Eur. J. Cancer Clin. Oncol.*, **20**, 1033.

COHEN, M.H., CREAVEN, P.J., FOSSIECK, B.F. & 5 others. (1977). Intensive chemotherapy of small cell bronchogenic carcinoma. *Cancer Treat. Rep.*, **61**, 349.

CORTES FUNES, H., DOMINGUEZ, P., PEREZ TORRUBIA, A., LANZOS, E., MENDEZ, M. & MENDIOLA, C. (1982). Treatment of small cell lung cancer with a combination of VP 16-213 and cyclophosphamide with *cis*-platin or radiotherapy. *Cancer Chemother. Pharmacol.*, **7**, 181.

CULLEN, M.H., MORGAN, D.A.L., RICHARDS, M.A., ROBINSON, M., WARD, M. & COX, D. (1983). Maintenance chemotherapy for small cell lung cancer – A randomised control trial. *2nd European Conference on Clinical Oncology and Cancer Nursing*, Amsterdam, November 1983. Abstract 10–17, p. 118.

GRECO, F.A., OLDHAM, R.K. & BUNN, P.A. (1981). Small cell lung cancer. *Clinical Oncology Monographs*, p. 261. Grune & Stratton: New York.

HANSEN, H.H. & ROTH, M. (1983). Small cell carcinoma of the lung. In *Recent Advances in Respiratory Medicine*, Flenley & Petty (eds) p. 193. Churchill Livingstone: Edinburgh.

IHDE, D.C., MAKUCH, R.W., CARNEY, D.W. & 4 others. (1981). Prognostic implications of stage of disease and sites of metastases in patients with small cell carcinoma of the lung treated with intensive combination chemotherapy. *Am. Rev. Respir. Dis.*, **123**, 500.

MEDICAL RESEARCH COUNCIL LUNG CANCER WORKING PARTY. (1979). Radiotherapy alone or with chemotherapy in the treatment of small-cell carcinoma of the lung. *Br. J. Cancer*, **40**, 1.

MONFARDINI, S., BRUNNER, K., CROWTHER, D. & 5 others. (1981). *Manual of Cancer Chemotherapy*, p. 17, UICC, Geneva.

OLDHAM, R.K. & GRECO, F.A. (1980). Small cell lung cancer. A curable disease. *Cancer Chemother. Pharmacol.*, **4**, 173.

POINTON, R.C.S., ASKILL, C., HUNTER, R.D. & WILKINSON, P.M. (1983). Treatment of advanced head and neck cancer using synchronous therapy with methotrexate and irradiation. *Clin. Radiol.*, **34**, 459.

SOUHAMI, R.L., HARPER, P.G., LINCH, D. & 6 others. (1983). High-dose cyclophosphamide with autologous marrow transplantation for small cell carcinoma of the bronchus. *Cancer Chemother. Pharmacol.*, **10**, 205.

SOUHAMI, R.L., GEDDES, D.M., SPIRO, S.G. & 5 others. (1984). Radiotherapy in small cell cancer of the lung treated with combination chemotherapy: A controlled trial. *Br. Med. J.*, **288**, 1643.

SPITTLE, M.F. (1978). Methotrexate and radiation. *Int. J. Radiat. Oncol. Biol. Phys.*, **4**, 103.

STRAUS, M.J., SELAWRY, O.S. & WALLACH, R.A. (1983). Chemotherapy in lung cancer. In *Lung Cancer Clinical Diagnosis and Treatment*, Straus (ed) p. 226. Grune and Stratton: New York.

THATCHER, N., HUNTER, R.D., JEGARAJAH, S. & 4 others. (1982*a*). Eleven-week course of sequential methotrexate, thoracic irradiation and moderate-dose cyclophosphamide for 'limited'-stage small cell bronchogenic carcinoma. A study from the Manchester Lung Tumour Group. *Lancet*, **i**, 1040.

THATCHER, N., WAGSTAFF, J., WILKINSON, P., PALMER, M. & CROWTHER, D. (1982*b*). Intermittent high-dose cyclophosphamide with and without prednisolone. A study of the relationship between toxicity, response and survival in metastatic lung cancer. *Cancer*, **50**, 1051.

THATCHER, N., HONEYBOURNE, D., WAGSTAFF, J. & 4 others. (1984*a*). Moderate to high dose cyclophosphamide and intercalacted corynebacterium parvum in patients with metastatic lung cancer. *Br. J. Dis. Chest*, **78**, 89.

THATCHER, N., JAMES, R.D., STEWARD, W.P. & 4 others. (1984*b*). Three months treatment with cyclophosphamide, in escalating dosage, followed by methotrexate and thoracic radiotherapy for small cell lung cancer. *Cancer* (in press).

WOODS, R.L. & LEVI, J.A. (1983). Chemotherapy for small cell lung cancer (SCLC). A randomised study of maintenance therapy with cyclophosphamide, adriamycin and vincristine (CAV) after remission induction with *cis*-platinum (*cis*-DDP), VP16–213 and radiotherapy. *2nd European Conference on Clinical Oncology and Cancer Nursing*, Amsterdam, November. Abstract 10–23, p. 119.